# Bis(1*H*-imidazole-κ*N* ^3^)bis­(2-methyl­benzoato-κ*O*)bis­(2-methyl­benzoic acid-κ*O*)copper(II)

**DOI:** 10.1107/S160053681102839X

**Published:** 2011-07-23

**Authors:** Sheng-Liang Ni, Ming-Xing Zhao, Hai-Xia Ge

**Affiliations:** a Department of Chemistry, Huzhou Teachers College, Huzhou, Zhejiang 313000, People’s Republic of China

## Abstract

The structure of the title compound, [Cu(C_8_H_7_O_2_)_2_(C_3_H_4_N_2_)_2_(C_8_H_8_O_2_)_2_], consists of centrosymmetric monomeric units, in which the Cu^II^ atom has a tetra­gonally distorted octa­hedral coordination involving two imidazole N atoms and two carboxyl­ate O atoms in the square plane [Cu—N = 1.964 (3) and Cu—O = 1.960 (2) Å] and 2-methyl­benzoic acid O atoms in axial sites [Cu—O = 2.753 (3) Å]. Within the complex, the carb­oxy­lic acid forms intra­molecular O—H⋯O hydrogen bonds, while the mol­ecules are assembled through N—H⋯O(carbox­yl) hydrogen bonds into chains extending along the *a*-axis direction. These chains are further linked by weak π–π inter­actions [centroid–centroid separation = 3.930 (2) Å].

## Related literature

For applications of transition metal complexes, see: Aakeröy & Seddon (1993 ▶). For the use of carboxyl­ate ligands in the construction of supra­molecular complexes, see: Moulton & Zaworotko (2001 ▶). For Cu—O/N distances in other tetra­gonally distorted octa­hedral copper(II) complexes, see: Bonamartini *et al.* (1993 ▶); Chen *et al.* (2010 ▶); Su *et al.* (1991 ▶). 
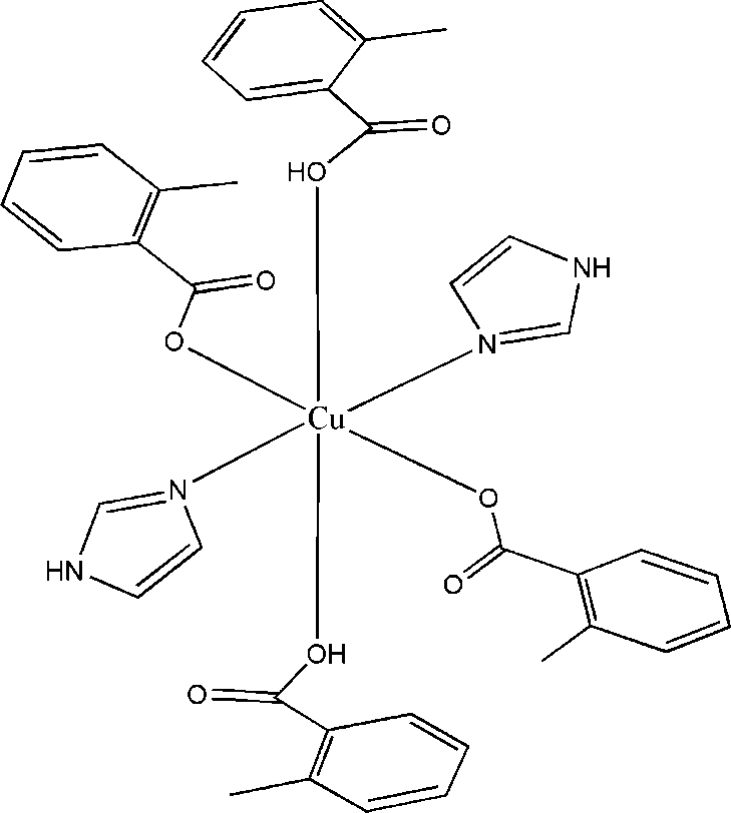


## Experimental

### 

#### Crystal data

[Cu(C_8_H_7_O_2_)_2_(C_3_H_4_N_2_)_2_(C_8_H_8_O_2_)_2_]
*M* *_r_* = 742.27
Monoclinic, 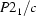

*a* = 8.0866 (16) Å
*b* = 12.193 (2) Å
*c* = 18.887 (4) Å
β = 101.90 (3)°
*V* = 1822.2 (6) Å^3^
*Z* = 2
Mo *K*α radiation
μ = 0.66 mm^−1^
*T* = 298 K
0.51 × 0.20 × 0.15 mm

#### Data collection

Rigaku R-AXIS RAPID CCD diffractometer
Absorption correction: multi-scan (*ABSCOR*; Higashi, 1995 ▶) *T*_min_ = 0.846, *T*_max_ = 0.901
17516 measured reflections
4135 independent reflections
2654 reflections with *I* > 2σ(*I*)
*R*_int_ = 0.035

#### Refinement

*R*[*F* ^2^ > 2σ(*F* ^2^)] = 0.046
*wR*(*F* ^2^) = 0.164
*S* = 1.14
4135 reflections
236 parameters
H atoms treated by a mixture of independent and constrained refinement
Δρ_max_ = 0.61 e Å^−3^
Δρ_min_ = −0.95 e Å^−3^

### 

Data collection: *RAPID-AUTO* (Rigaku, 1998 ▶); cell refinement: *RAPID-AUTO*; data reduction: *CrystalStructure* (Rigaku/MSC, 2004 ▶); program(s) used to solve structure: *SHELXS97* (Sheldrick, 2008 ▶); program(s) used to refine structure: *SHELXL97* (Sheldrick, 2008 ▶); molecular graphics: *SHELXTL* (Sheldrick, 2008 ▶); software used to prepare material for publication: *SHELXTL*.

## Supplementary Material

Crystal structure: contains datablock(s) global, I. DOI: 10.1107/S160053681102839X/zs2128sup1.cif

Structure factors: contains datablock(s) I. DOI: 10.1107/S160053681102839X/zs2128Isup2.hkl

Additional supplementary materials:  crystallographic information; 3D view; checkCIF report

## Figures and Tables

**Table 1:** Hydrogen-bond geometry (Å, °)

*D*—H⋯*A*	*D*—H	H⋯*A*	*D*⋯*A*	*D*—H⋯*A*
O3—H2⋯O2	0.85	1.67	2.516 (3)	168.9
N2—H1⋯O4^i^	0.89 (5)	1.97 (5)	2.786 (4)	152 (5)

Symmetry code: (i) 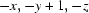.

**Fig. 1.**
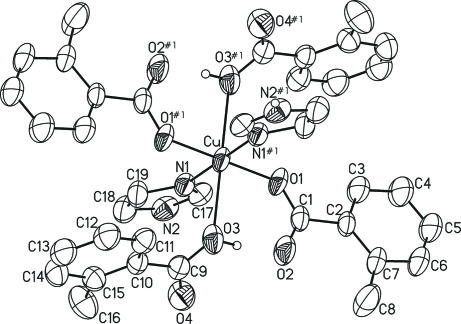
An ORTEP view of the title compound showing atom numbering, with displacement ellipsoids drawn at the 45% probability level. For symmetry code (#1): -x + 1, -y + 1, -z.

**Fig. 2.**
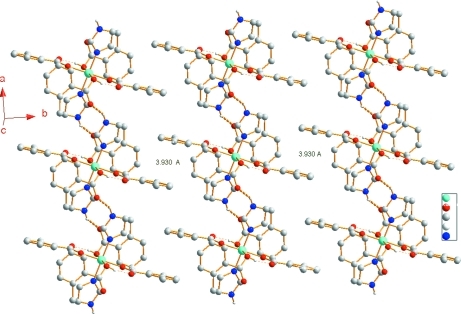
The structure of the title compound formed through hydrogen bonding and weak π–π packing interactions

## supplementary crystallographic information

### Comment

In the past decade, a variety of supramolecular architectures based on non-covalent intermolecular interactions such as hydrogen bonding, van der Waals forces and π–π stacking interactions have been achieved by using transition metal centers and organic ligands. Such complexes may have interesting structural topologies and have potential applications in catalysis, ion exchange, gas storage, and molecular-based magnetic materials (Aakeröy & Seddon, 1993). Carboxylate ligands have been commonly utilized as construction units to obtain a number of supramolecular complexes (Moulton & Zaworotko, 2001). We obtained the mononuclear title complex, [Cu(C_3_H_4_N_2_)_2_(C_8_H_7_O_2_)_2_(C_8_H_8_O_2_)_2_] from the reaction of imidazole and 2-methylbenzoic acid with CuCO_3_ in an aqueous ethanolic solution and its crystal structure is reported here.

The centrosymmetric title complex (Fig. 1) has tetragonally distorted octahedral stereochemistry, the coordination sphere about copper(II) comprising two two imidazole N donors and two carboxylate O donors in the square plane [Cu—N, 1.964 (3) Å; Cu—O, 1.960 (2) Å] and two O donors from the 2–methylbenzoic acid molecules in the axial sites [Cu—O, 2.753 (3) Å]. The Cu–O/N distances are similar to those found in other tetragonally distorted octahedral copper(II) complexes (Chen *et al.*, 2010, Bonamartini *et al.*, 1993, Su *et al.*, 1991). Within the complex the carboxylic acid forms intramolecular O—H···O hydrogen bonds (Table 1) while the molecules are assembled through N—H···O(carboxyl) hydrogen bonds into one-dimensional chains extending along the *a* cell direction (Fig. 2) and are further linked by weak π–π stacking [ring centroid separation, 3.930 (2) Å] giving layers extending across (110).

### Experimental

Freshly prepared CuCO_3_ was essential for an optimal synthesis. An aqueous solution of aqueous Na_2_CO_3_ (1.0 cm^3^; 1*M*) was added dropwise to 4 cm^3^ of a stirred aqueous solution of CuSO_4_. 5H_2_O (0.2490 g, 1.0 mmol), giving a precipitate of Cu(OH)_2–2x_(CO_3_)*_x_*.*y*H_2_O, which was centrifuged and washed with double distilled water until no SO_4_^2-^ anions were detected in the washings. The precipitate was subsequently added to a stirred solution of 2-methylbenzoic acid (0.5450 g, 4.0 mmol) and imidazole (0.069 g, 1.0 mmol) in 20 cm^3^ of  1:1 ethanol–water. The mixture was stirred for 1 h and the solid was removed by filtration, the resulting solution (pH = 4.10) was allowed to stand at room temperature, slow evaporation of the solvent forming blue block crystals after a week (yield: 43%).

### Refinement

All H-atoms bonded to C were positioned geometrically and refined using a riding model with C—H(aromatic) = 0.93 Å and *U*_iso_(H) = 1.2*U*_eq_(C) or C—H(methyl) = 0.96 Å and *U*_iso_(H) = 1.5*U*_eq_(C). H atoms attached to O atoms were found in a difference Fourier synthesis and were refined using a riding model, with O—H fixed as initially found and with *U*_iso_(H) values set at 1.5*U*_eq_(O).

### Crystal data

**Table:**

[Cu(C_8_H_7_O_2_)_2_(C_3_H_4_N_2_)_2_(C_8_H_8_O_2_)_2_]	*F*(000) = 774
*M**_r_* = 742.27	*D*_x_ = 1.353 Mg m^−^^3^
Monoclinic, *P*2_1_/*c*	Mo *K*α radiation, λ = 0.71073 Å
Hall symbol: -P 2ybc	Cell parameters from 25 reflections
*a* = 8.0866 (16) Å	θ = 3.0–27.4°
*b* = 12.193 (2) Å	µ = 0.66 mm^−^^1^
*c* = 18.887 (4) Å	*T* = 298 K
β = 101.90 (3)°	Block, blue
*V* = 1822.2 (6) Å^3^	0.51 × 0.20 × 0.15 mm
*Z* = 2	

### Data collection

**Table:**

Rigaku R-AXIS RAPID CCD diffractometer	4135 independent reflections
Radiation source: fine-focus sealed tube	2654 reflections with *I* > 2σ(*I*)
graphite	*R*_int_ = 0.035
ω scans	θ_max_ = 27.4°, θ_min_ = 3.0°
Absorption correction: multi-scan (*ABSCOR*; Higashi, 1995)	*h* = −9→10
*T*_min_ = 0.846, *T*_max_ = 0.901	*k* = −15→15
17516 measured reflections	*l* = −24→24

### Refinement

**Table:**

Refinement on *F*^2^	Primary atom site location: structure-invariant direct methods
Least-squares matrix: full	Secondary atom site location: difference Fourier map
*R*[*F*^2^ > 2σ(*F*^2^)] = 0.046	Hydrogen site location: inferred from neighbouring sites
*wR*(*F*^2^) = 0.164	H atoms treated by a mixture of independent and constrained refinement
*S* = 1.14	*w* = 1/[σ^2^(*F*_o_^2^) + (0.0599*P*)^2^ + 1.5834*P*] where *P* = (*F*_o_^2^ + 2*F*_c_^2^)/3
4135 reflections	(Δ/σ)_max_ < 0.001
236 parameters	Δρ_max_ = 0.61 e Å^−^^3^
0 restraints	Δρ_min_ = −0.95 e Å^−^^3^

### Special details

**Table:**

Geometry. All e.s.d.'s (except the e.s.d. in the dihedral angle between two l.s. planes) are estimated using the full covariance matrix. The cell e.s.d.'s are taken into account individually in the estimation of e.s.d.'s in distances, angles and torsion angles; correlations between e.s.d.'s in cell parameters are only used when they are defined by crystal symmetry. An approximate (isotropic) treatment of cell e.s.d.'s is used for estimating e.s.d.'s involving l.s. planes.
Refinement. Refinement of *F*^2^ against ALL reflections. The weighted *R*-factor *wR* and goodness of fit *S* are based on *F*^2^, conventional *R*-factors *R* are based on *F*, with *F* set to zero for negative *F*^2^. The threshold expression of *F*^2^ > σ(*F*^2^) is used only for calculating *R*-factors(gt) *etc*. and is not relevant to the choice of reflections for refinement. *R*-factors based on *F*^2^ are statistically about twice as large as those based on *F*, and *R*- factors based on ALL data will be even larger.

### Fractional atomic coordinates and isotropic or equivalent isotropic displacement parameters (Å^2^)

**Table:**

	*x*	*y*	*z*	*U*_iso_*/*U*_eq_	
Cu	0.5000	0.5000	0.0000	0.0496 (2)	
O1	0.4213 (3)	0.64617 (17)	0.02095 (14)	0.0596 (6)	
O2	0.2854 (4)	0.6947 (2)	−0.08906 (16)	0.0783 (8)	
C1	0.3427 (4)	0.7153 (2)	−0.0237 (2)	0.0513 (8)	
C2	0.3180 (4)	0.8272 (2)	0.00562 (19)	0.0511 (8)	
C3	0.3655 (6)	0.8403 (3)	0.0799 (2)	0.0726 (11)	
H3A	0.4116	0.7813	0.1085	0.087*	
C4	0.3455 (7)	0.9400 (4)	0.1122 (3)	0.0979 (17)	
H4A	0.3769	0.9477	0.1622	0.117*	
C5	0.2799 (7)	1.0264 (3)	0.0705 (3)	0.0948 (16)	
H5A	0.2671	1.0938	0.0918	0.114*	
C6	0.2329 (7)	1.0143 (3)	−0.0021 (3)	0.0848 (14)	
H6A	0.1882	1.0745	−0.0297	0.102*	
C7	0.2485 (5)	0.9156 (3)	−0.0373 (2)	0.0640 (10)	
C8	0.1914 (8)	0.9123 (4)	−0.1184 (2)	0.1054 (18)	
H8A	0.1491	0.9831	−0.1356	0.158*	
H8B	0.1035	0.8586	−0.1314	0.158*	
H8C	0.2852	0.8931	−0.1399	0.158*	
O3	0.3580 (3)	0.51870 (19)	−0.14514 (16)	0.0670 (7)	
H2	0.3217	0.5742	−0.1255	0.080*	
O4	0.0849 (4)	0.4803 (2)	−0.16537 (18)	0.0801 (9)	
C9	0.2279 (5)	0.4565 (3)	−0.17091 (18)	0.0536 (8)	
C10	0.2697 (4)	0.3551 (3)	−0.20755 (17)	0.0510 (8)	
C11	0.4218 (5)	0.3531 (3)	−0.2315 (2)	0.0611 (9)	
H11A	0.4940	0.4132	−0.2227	0.073*	
C12	0.4669 (6)	0.2638 (3)	−0.2680 (2)	0.0715 (11)	
H12A	0.5672	0.2642	−0.2848	0.086*	
C13	0.3628 (6)	0.1750 (3)	−0.2791 (2)	0.0772 (12)	
H13A	0.3924	0.1143	−0.3037	0.093*	
C14	0.2145 (6)	0.1743 (3)	−0.2545 (2)	0.0746 (11)	
H14A	0.1468	0.1119	−0.2616	0.089*	
C15	0.1622 (5)	0.2648 (3)	−0.21914 (19)	0.0599 (9)	
C16	−0.0046 (6)	0.2574 (4)	−0.1956 (3)	0.0896 (14)	
H16A	−0.0250	0.3245	−0.1723	0.134*	
H16B	−0.0014	0.1974	−0.1624	0.134*	
H16C	−0.0935	0.2455	−0.2372	0.134*	
N1	0.2954 (3)	0.4336 (2)	0.02226 (15)	0.0499 (6)	
N2	0.0739 (4)	0.4079 (3)	0.06931 (18)	0.0614 (8)	
H1	−0.003 (6)	0.426 (4)	0.095 (3)	0.101 (16)*	
C17	0.1960 (5)	0.4773 (3)	0.0623 (2)	0.0553 (8)	
H17A	0.2098	0.5470	0.0828	0.066*	
C18	0.0930 (5)	0.3148 (3)	0.0321 (2)	0.0677 (10)	
H18A	0.0249	0.2527	0.0272	0.081*	
C19	0.2311 (5)	0.3312 (3)	0.0036 (2)	0.0606 (9)	
H19A	0.2757	0.2808	−0.0243	0.073*	

### Atomic displacement parameters (Å^2^)

**Table:**

	*U*^11^	*U*^22^	*U*^33^	*U*^12^	*U*^13^	*U*^23^
Cu	0.0522 (3)	0.0276 (3)	0.0718 (4)	0.0049 (2)	0.0195 (3)	0.0006 (2)
O1	0.0704 (16)	0.0301 (11)	0.0816 (16)	0.0099 (11)	0.0236 (13)	0.0000 (11)
O2	0.109 (2)	0.0432 (14)	0.0782 (19)	0.0115 (14)	0.0089 (16)	−0.0148 (13)
C1	0.0493 (18)	0.0308 (15)	0.077 (2)	0.0002 (13)	0.0212 (17)	−0.0053 (15)
C2	0.0526 (18)	0.0305 (15)	0.071 (2)	−0.0009 (13)	0.0140 (16)	−0.0058 (14)
C3	0.094 (3)	0.0404 (19)	0.076 (3)	0.0172 (19)	−0.001 (2)	−0.0058 (17)
C4	0.136 (4)	0.062 (3)	0.082 (3)	0.028 (3)	−0.009 (3)	−0.026 (2)
C5	0.123 (4)	0.043 (2)	0.105 (4)	0.024 (2)	−0.006 (3)	−0.026 (2)
C6	0.111 (4)	0.038 (2)	0.097 (3)	0.019 (2)	0.002 (3)	−0.005 (2)
C7	0.081 (3)	0.0335 (16)	0.075 (2)	0.0052 (16)	0.011 (2)	−0.0013 (16)
C8	0.178 (6)	0.059 (3)	0.074 (3)	0.023 (3)	0.012 (3)	0.007 (2)
O3	0.0695 (17)	0.0451 (14)	0.0900 (19)	−0.0034 (12)	0.0250 (15)	−0.0162 (12)
O4	0.0636 (17)	0.0740 (19)	0.104 (2)	0.0116 (14)	0.0206 (16)	−0.0226 (16)
C9	0.062 (2)	0.0457 (17)	0.0527 (19)	0.0034 (16)	0.0119 (16)	0.0010 (15)
C10	0.062 (2)	0.0423 (17)	0.0481 (17)	0.0031 (15)	0.0102 (15)	−0.0023 (14)
C11	0.069 (2)	0.052 (2)	0.064 (2)	0.0015 (17)	0.0177 (18)	−0.0044 (17)
C12	0.076 (3)	0.070 (3)	0.071 (2)	0.015 (2)	0.023 (2)	−0.009 (2)
C13	0.097 (3)	0.060 (2)	0.071 (3)	0.017 (2)	0.009 (2)	−0.017 (2)
C14	0.094 (3)	0.052 (2)	0.071 (3)	−0.009 (2)	0.003 (2)	−0.0131 (19)
C15	0.068 (2)	0.055 (2)	0.054 (2)	−0.0033 (17)	0.0062 (17)	−0.0018 (16)
C16	0.080 (3)	0.094 (3)	0.097 (3)	−0.029 (3)	0.025 (3)	−0.017 (3)
N1	0.0511 (15)	0.0373 (14)	0.0620 (17)	0.0039 (11)	0.0133 (13)	0.0012 (12)
N2	0.0509 (17)	0.066 (2)	0.070 (2)	0.0089 (15)	0.0193 (15)	0.0117 (16)
C17	0.058 (2)	0.0468 (19)	0.062 (2)	0.0105 (15)	0.0143 (17)	0.0008 (15)
C18	0.060 (2)	0.051 (2)	0.095 (3)	−0.0049 (17)	0.023 (2)	0.005 (2)
C19	0.062 (2)	0.0398 (17)	0.083 (3)	−0.0035 (15)	0.0226 (19)	−0.0059 (17)

### Geometric parameters (Å, °)

**Table:**

Cu—O1^i^	1.960 (2)	O4—C9	1.217 (4)
Cu—O1	1.960 (2)	C9—C10	1.489 (5)
Cu—O3^i^	2.753 (3)	C10—C15	1.392 (5)
Cu—O3	2.753 (3)	C10—C11	1.396 (5)
Cu—N1^i^	1.964 (3)	C11—C12	1.377 (5)
Cu—N1	1.964 (3)	C11—H11A	0.9300
O1—C1	1.267 (4)	C12—C13	1.360 (6)
O2—C1	1.252 (4)	C12—H12A	0.9300
C1—C2	1.501 (4)	C13—C14	1.373 (6)
C2—C3	1.385 (5)	C13—H13A	0.9300
C2—C7	1.395 (5)	C14—C15	1.400 (5)
C3—C4	1.385 (5)	C14—H14A	0.9300
C3—H3A	0.9300	C15—C16	1.507 (6)
C4—C5	1.356 (6)	C16—H16A	0.9600
C4—H4A	0.9300	C16—H16B	0.9600
C5—C6	1.353 (7)	C16—H16C	0.9600
C5—H5A	0.9300	N1—C17	1.325 (4)
C6—C7	1.393 (5)	N1—C19	1.371 (4)
C6—H6A	0.9300	N2—C17	1.327 (5)
C7—C8	1.506 (6)	N2—C18	1.361 (5)
C8—H8A	0.9600	N2—H1	0.89 (5)
C8—H8B	0.9600	C17—H17A	0.9300
C8—H8C	0.9600	C18—C19	1.350 (5)
O3—C9	1.307 (4)	C18—H18A	0.9300
O3—H2	0.8519	C19—H19A	0.9300
			
O1^i^—Cu—O1	180.00 (15)	C15—C10—C9	122.4 (3)
O1^i^—Cu—N1^i^	90.49 (10)	C11—C10—C9	117.7 (3)
O1—Cu—N1^i^	89.51 (10)	C12—C11—C10	121.2 (4)
O1^i^—Cu—N1	89.51 (10)	C12—C11—H11A	119.4
O1—Cu—N1	90.49 (10)	C10—C11—H11A	119.4
N1^i^—Cu—N1	180.0	C13—C12—C11	119.2 (4)
C1—O1—Cu	127.6 (2)	C13—C12—H12A	120.4
O2—C1—O1	123.8 (3)	C11—C12—H12A	120.4
O2—C1—C2	119.7 (3)	C12—C13—C14	120.6 (4)
O1—C1—C2	116.5 (3)	C12—C13—H13A	119.7
C3—C2—C7	119.6 (3)	C14—C13—H13A	119.7
C3—C2—C1	116.6 (3)	C13—C14—C15	121.8 (4)
C7—C2—C1	123.8 (3)	C13—C14—H14A	119.1
C2—C3—C4	121.0 (4)	C15—C14—H14A	119.1
C2—C3—H3A	119.5	C10—C15—C14	117.3 (4)
C4—C3—H3A	119.5	C10—C15—C16	124.7 (4)
C5—C4—C3	119.6 (4)	C14—C15—C16	118.0 (4)
C5—C4—H4A	120.2	C15—C16—H16A	109.5
C3—C4—H4A	120.2	C15—C16—H16B	109.5
C6—C5—C4	119.9 (4)	H16A—C16—H16B	109.5
C6—C5—H5A	120.1	C15—C16—H16C	109.5
C4—C5—H5A	120.1	H16A—C16—H16C	109.5
C5—C6—C7	123.0 (4)	H16B—C16—H16C	109.5
C5—C6—H6A	118.5	C17—N1—C19	105.7 (3)
C7—C6—H6A	118.5	C17—N1—Cu	126.5 (2)
C6—C7—C2	117.1 (4)	C19—N1—Cu	127.8 (2)
C6—C7—C8	118.0 (4)	C17—N2—C18	108.3 (3)
C2—C7—C8	124.9 (3)	C17—N2—H1	121 (3)
C7—C8—H8A	109.5	C18—N2—H1	131 (3)
C7—C8—H8B	109.5	N1—C17—N2	110.6 (3)
H8A—C8—H8B	109.5	N1—C17—H17A	124.7
C7—C8—H8C	109.5	N2—C17—H17A	124.7
H8A—C8—H8C	109.5	C19—C18—N2	105.8 (3)
H8B—C8—H8C	109.5	C19—C18—H18A	127.1
C9—O3—H2	107.4	N2—C18—H18A	127.1
O4—C9—O3	122.4 (3)	C18—C19—N1	109.5 (3)
O4—C9—C10	123.3 (3)	C18—C19—H19A	125.2
O3—C9—C10	114.3 (3)	N1—C19—H19A	125.2
C15—C10—C11	119.9 (3)		

Symmetry codes: (i) −*x*+1, −*y*+1, −*z*.

### Hydrogen-bond geometry (Å, °)

**Table:**

*D*—H···*A*	*D*—H	H···*A*	*D*···*A*	*D*—H···*A*
O3—H2···O2	0.85	1.67	2.516 (3)	168.9
N2—H1···O4^ii^	0.89 (5)	1.97 (5)	2.786 (4)	152 (5)

Symmetry codes: (ii) −*x*, −*y*+1, −*z*.
